# Perceived barriers to healthy eating and self-efficacy among Indian adults at high risk of type 2 diabetes: a secondary analysis of the K-DPP study

**DOI:** 10.3389/fnut.2026.1777691

**Published:** 2026-05-25

**Authors:** Mayuko McMillin, Tilahun Haregu, Panniyammakal Jeemon, Kavumpurathu Raman Thankappan, Brian Oldenburg, Yingting Cao

**Affiliations:** 1 School of Allied Health, Human Services and Sport, La Trobe University, Melbourne, VIC, Australia; 2 Non-Communicable Diseases and Implementation Science Lab, Baker Heart and Diabetes Institute, Melbourne, VIC, Australia; 3 School of Psychology and Public Health, La Trobe University, Melbourne, VIC, Australia; 4 Sree Chitra Tirunal Institute for Medical Sciences and Technology, Trivandrum, India; 5 Department of Public Health, Amrita Institute of Medical Sciences, Amrita Vishwa Vidyapeetham, Kochi, Kerala, India; 6 Victorian Centre for Virtual Health Research, Northern Health, Melbourne, VIC, Australia

**Keywords:** dietary self-efficacy, fruit and vegetable intake, India, perceived barrier, type 2 diabetes risk

## Abstract

**Introduction:**

Low fruit and vegetable (FV) consumption is a major contributor to the rising burden of non-communicable diseases, yet intake in India remains far below recommended levels. Although structural barriers to healthy eating are widely documented, little is known about how perceived barriers and dietary self-efficacy influence FV intake in high-risk populations, especially in a country like India.

**Methods:**

This study analyzed longitudinal data from 1,007 Indian adults participating in the Kerala Diabetes Prevention Program (K-DPP) in order to examine the effects of perceived barriers and dietary self-efficacy on FV intake over 9 years. Linear mixed-effects models were used to evaluate five perceived barrier domains and four dietary self-efficacy items in relation to FV intake (g/day) across four waves. Predictors were modeled categorically using the lowest-barrier and highest-efficacy groups as reference groups, with adjustment for demographic and behavioral covariates.

**Results:**

Higher perceived barriers related to availability (β = −53.9 g/day, 95% CI [−80.7, −27.3], *p* < 0.001) and cost (β = −25.3 g/day, 95% CI [−46.6, −4.0], *p* < 0.02), along with low dietary self-efficacy in adapting to different tastes (β = −43.0 g/day, 95% CI [−76.6, −9.4], *p* = 0.01) were significantly associated with lower FV intake.

**Discussion:**

These findings highlight that perceived structural constraints, particularly limited availability and affordability, are key correlates of low FV intake in this population, and may inform interventions that prioritize improving access to FVs as a foundation for sustainable dietary improvement.

## Introduction

Insufficient fruit and vegetable (FV) intake is a major, modifiable contributor to the global burden of non-communicable diseases, including in low- and middle-income countries (LMICs) (1, 2). In India, reported FV intake remains substantially below recommended levels- 400 g/day internationally and 350 g/day nationally (3, 4)- with average intakes of only 160 g in rural areas, 184 g in urban areas, and a national median of 200 g (5, 6). Despite nationwide nutritional campaigns and growing public awareness of the health benefits of FV consumption (7, 8), this persistent gap is shaped by food-system pressures and sociocultural influences that reinforce structural and psychosocial barriers to adequate intake (9–11).

In India, structural barriers to healthy eating are substantial, encompassing economic and food-system constraints. More than 60% of rural Indians cannot afford a nutritious diet, limiting the ability to achieve adequate FV intake, and achieving such a diet can consume nearly one-fifth of household income in comparable LMIC contexts (12, 13). These affordability pressures can influence household purchasing patterns, often encouraging reliance on cheaper, energy-dense foods and limiting the inclusion of nutrient-rich options (14, 15). This cost burden largely arises from systemic food-supply limitations; inadequate cold-storage capacity and inefficient distribution systems contribute to 20–40% post-harvest losses, elevating retail prices of fresh produce (10, 16). Together, these structural constraints are likely to shape dietary behaviors and highlight the importance of understanding how they are perceived and navigated at the individual level.

Against this backdrop, Kerala represents a particularly informative setting in which to examine these dynamics. Despite relatively higher literacy levels and more favorable health indicators than other Indian states (17), Kerala faces a high and increasing burden of non-communicable diseases, including type 2 diabetes (18–20). This pattern has been linked to broader processes such as urbanization and economic development, reflecting an ongoing epidemiological and nutrition transition. While structural determinants of suboptimal diet are well documented globally, far less is known about how individuals perceive and internalize these constraints, and how such perceptions influence dietary decision-making. These processes may operate differently in populations undergoing rapid social, dietary, and lifestyle change; examining psychosocial correlates of fruit and vegetable intake in Kerala may therefore provide insights relevant to similar transitional settings.

Perceived barriers (PBs)- individuals' subjective beliefs about obstacles to healthy eating- can shape dietary behavior regardless of their alignment with objective reality (21). Within the Health Belief Model (HBM), PBs represent a core construct shaping how individuals interpret challenges within their environments and determine the feasibility of adopting health-promoting behaviors (22). Importantly though, PBs do not form in isolation; they are socially patterned by broader socioeconomic and cultural contexts, which influence both the types of barriers perceived and the extent to which they impede behavior (23, 24). In LMICs, structural constraints, such as affordability, inconsistent supply, and limited access, are frequently identified as dominant barriers to FV intake (8, 25, 26). Evidence from India further underscores the role of household decision-making, family responsibilities, and social norms on dietary behavior (11), pointing to more complex interactions between structural and psychosocial factors. In high-income countries, barriers more often stem from time constraints, convenience-driven food environments, and limited cooking time and skills, despite abundant food availability (27–29). Overall, this evidence illustrates that PBs reflect a dynamic interplay between contextual conditions and individual-level interpretations, highlighting the importance of considering both when examining FV consumption.

Self-efficacy, another core construct later incorporated into the HBM, is defined as confidence in one's ability to perform a behavior (22). In the contexts of eating behaviors, dietary self-efficacy (DSE) reflects the capacity to initiate and sustain healthy eating practices, thereby influencing motivation, persistence, and responses to barriers. Empirical evidence consistently links higher DSE with greater FV intake across diverse populations (30–32). DSE may further buffer challenges such as cost, social pressure, and limited family support (33, 34), and may mediate the relationship between nutritional knowledge and dietary behavior, underscoring its conceptual importance (35). Globally, interventions integrating nutrition education and/or peer or community support have demonstrated improvements in DSE, reductions in PBs, and subsequent increases in FV intake (36, 37). However, emerging evidence indicates that confidence alone rarely leads to lasting change when supportive environments are lacking, as structural factors such as cost and availability often exert stronger and persistent effects (38).

Although PBs and DSE are conceptually and empirically well-established, studies jointly examining their influences on FV intake remain limited, particularly among adults in LMICs. Much of the existing research is cross-sectional, focused on adolescents, or treats FV intake as a single aggregated outcome. Consequently, longitudinal evidence on how specific PB domains and DSE components influence FV intake in adults is scarce. Moreover, few studies have assessed their relative contributions within a unified analytical framework, despite their complementary roles under behavioral theory. Dietary behavior can arise from individual-level cognitions (e.g., self-efficacy) alongside perceptions of broader environmental and social conditions (e.g., cost, availability, and family context), shaped through dynamic interactions across these levels (33, 39). However, such multilevel integration remains underexplored in LMIC research, leaving critical gaps in understanding how PBs and DSE operate concurrently or whether their effects vary among adults at elevated risk of non-communicable diseases. Addressing these gaps is crucial to developing contextually relevant, structurally informed interventions in India and other LMIC settings.

Accordingly, the present study undertakes a secondary analysis of data from the Kerala Diabetes Prevention Program (K-DPP), a community-based cohort of adults at high risk of developing T2DM (18). The aims were to: 1) examine nine-year trends in fruit, vegetables, and combined FV intake; 2) evaluate levels of specific PB domains and DSE items, and their cross-sectional associations with FV intake; and 3) assess longitudinal associations of PBs and DSE with fruit, vegetable, and combined FV intake over four waves.

## Method

### Study design

This secondary analysis used data from the K-DPP, a cluster-randomized controlled trial with longitudinal follow-up conducted in Kerala, India, between 2013 and 2023 (40, 41). The original trial recruited 1,007 adults aged 30–60 years at high risk of type 2 diabetes mellitus (Indian Diabetes Risk Score ≥60) from 60 polling areas and randomized them to intervention or control arms. Data were collected at four time points: baseline, 1-year, 2-year, and 9-year follow-up. Eligibility criteria excluded individuals with prior diabetes diagnosis, pregnancy, chronic illness, use of glucose-lowering medication, or diabetes confirmed at baseline by oral glucose tolerance testing. Further details of the trial protocol have been published elsewhere (40, 41). All participants from both trial arms were retained in this secondary analysis, as the present study did not aim to evaluate intervention effects.

### Dietary assessment

Dietary intake at baseline, 1-year, and 2-year follow-ups was assessed using a food frequency questionnaire (FFQ), which was adapted from the PROLIFE study previously conducted in Kerala (20, 42, 43). Participants reported usual intake over the preceding 12 months, including consumption frequency (daily, weekly, or monthly) and portion size. Reported intakes were converted into gram-per-day estimates using context-specific portion weights. Vegetable intake was categorized as tubers and non-tuber vegetables, and fruit consumption was separately reported for common types, including banana, water apple, guava, pineapple, watermelon, mango, jackfruit, apple, grape, pomegranate, and orange. At the 9-year follow-up, dietary data were collected using a shortened questionnaire capturing weekly frequency and daily servings of total fruit and total vegetable intake without disaggregation by specific types. To enable comparability across waves, serving-based responses at year 9 were converted into approximate gram-per-day estimates using a standard serving size of 80 g, consistent with widely used public health conventions (4, 42, 43). This approach was used to preserve longitudinal comparability and does not imply strict equivalence between dietary assessment instruments.

### Exposure variables

Perceived barriers (PBs) and dietary self-efficacy (DSE) were assessed using self-administered questionnaires that were culturally adapted for the K-DPP context (38, 40). PBs were captured using 5-point Likert-type scales across five domains: availability, taste, time, cost, and family support. Each domain was assessed using a single-item measure representing commonly reported and conceptually distinct barriers to healthy eating, identified *a priori* based on theoretical and empirical relevance to this population. Together, these items support the face and content validity of the PB measures in this context.

DSE was assessed using a 4-point Likert-type scale across six items reflecting confidence in performing specific healthy dietary behaviors. PB and DSE variables were retained as ordinal categorical predictors in longitudinal models to avoid imposing linearity assumptions on Likert-type scales, as the intervals between response options may not be equal. This approach also allows for the detection of potential non-linear or threshold effects, which are plausible in the context of psychosocial constructs. For descriptive and bivariate analyses, responses were collapsed into simplified categories to improve interpretability. Detailed item wording and response scales are provided in Supplementary Table 1.

It should be noted that these instruments have not undergone formal psychometric validation in this population. The DSE scale demonstrated excellent internal consistency (Cronbach's α = 0.949); however, this could not be assessed for PB domains because each was measured using a single item.

### Outcome variables

Primary outcomes were daily intake (g/day) of fruit, vegetables (including tubers), and combined fruit and vegetable intake (including tubers). These outcomes were derived from self-reported dietary data collected at each assessment wave.

### Covariates

Sociodemographic and behavioral variables were measured using WHO STEPwise surveillance instruments adapted for the local context (41). Covariates included in the fully adjusted model were selected *a priori* based on theoretical relevance and prior literature, and comprised age, sex, marital status, education, household expenditure, physical activity, sleep duration, sedentary behavior, and family history of diabetes. Trial arm was also included as a covariate in all multivariable models to account for intervention effects.

### Statistical analysis

Analyses were conducted using data from all waves (baseline, year 1, year 2, and year 9), with retention rates exceeding 85% across follow-ups. Participants from both trial arms were included to maximize longitudinal power, with clustering addressed analytically. Participant characteristics, FV intake across time points and sociodemographic groups, and baseline distributions of PB and DSE were summarized using descriptive statistics. Bivariate associations were examined using Kruskal–Wallis tests for PB domains (recoded as high, low, or neither) and Mann–Whitney U tests for dichotomized DSE categories (high vs. low), with Bonferroni-adjusted *post hoc* comparisons as appropriate.

Longitudinal associations of PBs and DSE with FV intake were analyzed using linear mixed-effects models. Participant ID was specified as a random intercept to account for within-person correlation, and timepoint was modeled as a repeated measure to capture longitudinal change. Random slopes for time were not included due to the limited number of observations per participant and the uneven spacing of follow-up assessments (1, 2, and 9 years), which may reduce the reliability of individual trajectory estimates and increase the risk of model instability. Accordingly, the model was specified to estimate overall associations across the study population. Study cluster was included as a fixed effect to account for baseline clustering and intervention-related differences inherent to the study design. As analyses were guided by *a priori* hypotheses and focused on a limited set of theoretically relevant exposures, formal correction for multiple comparisons was not applied.

Statistical significance was set at *p* < 0.05, and normality was assessed using the Shapiro-Wilk test. All analyses were conducted using IBM SPSS Statistics (version 30).

## Results

A total of 1,007 adults (47.2% women) at high risk of type 2 diabetes (T2DM) were included in the analysis. Baseline sociodemographic, anthropometric, and clinical characteristics have been reported in detail elsewhere (37). Overall, the cohort was predominantly in mid-adulthood (median age 46 years), with a high prevalence of family history of diabetes (47.9%) and overweight or obesity status (68.5%).

Fruit intake declined steadily from baseline to year 2, followed by a marked decrease in year 9 (baseline: 96.8 g/day [IQR 126.8]; year 9: 57.1 g/day [IQR 34.3]). Dietary intake at year 9 was assessed using a different methodology from earlier time points. In contrast, vegetable intake increased over time (102.9 g/day [IQR 90.1] to 160.0 g/day [IQR 80.0]), resulting in relatively stable combined fruit and vegetable (FV) intake (224.6 g/day [IQR 168.5] at baseline vs. 240.0 g/day [IQR 97.1] at year 9). The median FV intake across all time points was 234.3 g/day. Men consistently reported higher FV intakes than women, particularly for fruit (baseline medians: 118.9 g/day vs. 76.6 g/day), although temporal patterns were similar across sexes. At baseline, higher fruit intake was associated with male sex, higher educational attainment, greater household expenditure, leisure-time physical activity, and a family history of diabetes (all *p* < 0.005). Vegetable intake showed fewer sociodemographic associations, differing modestly by marital status and household size only (both *p* < 0.05) (Table 1).

**Table 1:** Fruit and vegetable intake by demographic/behavioral groups at baseline.

Variable	Categories	Fruit Median g/day	MWU or KW *P*-value	Veg/Tuber Median g/day	MWU or KW *P*-value
Sex	Male	118.9 (142.3)	< 0.001	102.9 (88.8)	0.117
	Female	76.6 (102.1)		97.1 (85.5)	
Marital status	Married	97.2 (127.5)	0.022	102.9 (90.1)	0.011
	Separated/Divorced	52.5 (76.3)		48.6 (59.3)	
	Widowed	71.8 (160.8)		91.4 (84.3)	
	Single	82.5 (125.4)		97.1 (28.6)	
Age	< =45	100.1 (131.8)	0.437	102.9 (90.1)	0.794
	45 < 53	94.3 (133.3)		102.9 (87.4)	
	53+	94.2 (112.9)		97.1 (91.4)	
Household size	1–2 people	71.8 (113.9)	0.261	91.4 (114.6)	0.018
	3 people	96.5 (132.7)		102.9 (83.1)	
	4 people	99.2 (127.3)		102.9 (87.4)	
	5 people +	95.3 (129.0)		102.9 (91.1)	
Education category	Up to primary (> 6 years)	69.1 (105.7)	< 0.001	97.1 (85.8)	0.189
	Middle school (7–8 years)	91.3 (128.1)		102.9 (97.1)	
	Secondary (9–10 years)	97.3 (125.7)		111.4 (89.8)	
	Higher secondary (11–years)	97.4 (112.2)		102.9 (83.1)	
	Vocational, higher ed (12 +)	126.1 (158.9)		114.3 (86.1)	
Monthly household expenditure (INR)	Low (5,500 or less)	92.2 (106.9)	0.002	102.9 (97.1)	0.478
	Med (5,500–9,500)	88.4 (129.0)		102.9 (83.1)	
	High (9,500+)	110.5 (137.2)		102.9 (90.1)	
Sedentary hours per day	1 h or less	94.8 (117.3)	0.483	102.9 (91.4)	0.199
	1–3 h	102.9 (132.0)		105.7 (87.4)	
	More than 3 h	96.0 (139.7)		96.6 (80.3)	
Leisure PA	No	90.0 (117.9)	< 0.001	102.9 (90.1)	0.117
	Yes	131.8 (146.5)		114.3 (86.1)	
Alcohol	No	94.2 (122.3)	0.009	102.9 (90.1)	0.638
	Yes	114.1 (144.3)		102.9 (89.1)	
Smoking	Smoking	102.9 (166.2)	0.453	91.4 (80.0)	0.153
	Smokeless	125.4 (134.6)		114.3 (114.3)	
	Both	77.5 (110.6)		160.0 (144.6)	
	None	95.3 (123.0)		102.9 (88.8)	
Waist	< 90 cm	86.6 (112.3)	0.001	97.1 (90.1)	0.781
	90 cm+	105.7 (142.2)		102.9 (90.1)	
BMI	Underweight (< 18.5)	76.2 (139.0)	0.714	97.1 (80.0)	0.250
	Normal (18.5 = < 25)	96.8 (125.8)		102.9 (87.4)	
	Overweight (25 = < 30)	99.7 (130.3)		97.1 (91.4)	
	Obese (30 +)	96.0 (137.2)		131.4 (88.8)	
Family history of diabetes	No	63.9 (114.2)	< 0.001	102.9 (88.8)	0.616
	Yes	104.1 (131.3)		102.9 (90.1)	

Values are presented as median (interquartile range, IQR) to reflect non-parametric data. Group differences in fruit and vegetable/tuber intake were examined using Mann-Whitney U tests for binary variables and Kruskal-Wallis tests for variables with more than two categories. Intake values represent grams per day based on self-reported food frequency questionnaire data at baseline.

Table 2 presents baseline distributions of perceived barriers (PBs) and dietary self-efficacy (DSE). Taste- (54%) and cost-related (46%) PBs were most frequently reported, whereas time-related PBs were least common (18%). DSE levels were generally high across domains (60–73%), although confidence was lowest in situations where healthy foods were unavailable. In unadjusted analyses at baseline (Supplementary Table 2), fruit intake differed by cost- and taste-related PBs, while vegetable intake differed by taste- and family-related PBs (all *p* < 0.05). No consistent unadjusted associations were observed across DSE domains.

**Table 2:** Distribution of perceived barrier and dietary self-efficacy categories at baseline.

Predictor	Category	Level	Total *N* (%)
Perceived barriers	Availability	High barrier	339 (33.7%)
		Low barrier	661 (65.6%)
		Neither	7 (0.7%)
	Taste	High barrier	547 (54.3%)
		Low barrier	403 (40.0%)
		Neither	57 (5.7%)
	Time	High barrier	177 (17.6%)
		Low barrier	802 (79.7%)
		Neither	27 (2.7%)
	Cost	High barrier	458 (45.5%)
		Low barrier	519 (51.5%)
		Neither	30 (3.0%)
	Family	High barrier	234 (23.3%)
		Low barrier	746 (74.1%)
		Neither	27 (2.7%)
Dietary self-efficacy	Cooking	Lower DSE	275 (27.3)
		Higher DSE	732 (72.7)
	Taste	Lower DSE	303 (30.1)
		Higher DSE	703 (69.9)
	Eat_less	Lower DSE	333 (33.1)
		Higher DSE	673 (66.9)
	Family_support	Lower DSE	360 (35.8)
		Higher DSE	646 (64.2)
	Canteen	Lower DSE	411 (40.8)
		Higher DSE	596 (59.2)
	Around_me	Lower DSE	401 (39.9)
		Higher DSE	604 (60.1)

Perceived barrier (PB) categories were derived from self-reported survey items rated on a 5-point Likert scale (1 = Strongly agree to 5 = Strongly disagree). “High barrier” corresponds to responses of *Strongly agree* or *Agree* (indicating higher perceived difficulty in eating healthily), “Low barrier” corresponds to *Strongly disagree* or *Disagree*, and “Neither” represents *Neither agree nor disagree* responses. Percentages are calculated within each PB domain at baseline. Dietary self-efficacy (DSE) categories were derived from self-reported survey items rated on a 4-point Likert scale (1 = Very uncertain to 4 = Very certain). “Lower DSE” corresponds to responses of *Very uncertain* or *Rather uncertain*, while “Higher DSE” corresponds to *Rather certain* or *Very certain*. Percentages are calculated within each DSE domain at baseline.

The results of adjusted linear mixed models for combined FV intake, fruit intake, and vegetable intake are presented in Table 3. PBs related to availability, cost, and taste, as well as taste-related DSE demonstrated significant overall (omnibus) associations with combined FV intake. Availability PBs were the strongest and most consistent predictors, corresponding to 53.9 g/day lower intake in the highest PB group (95% CI [−80.7, −27.3], *p* < 0.001). Cost-related PBs followed a weaker, non-monotonic pattern, with only one category reaching significance (β = −25.3 g/day, 95% CI [−46.6, −4.0], *p* = 0.020). Contrary to expectations, FV intake increased across taste-related PB groups, with participants in the highest PB group consuming on average 41.6 g/day more FV than those in the lowest group (95% CI [12.4, 70.7], *p* = 0.005). Among DSE domains, only taste-related self-efficacy was significantly associated with FV intake: participants in the lowest confidence category consumed 43.0 g/day less than those with the highest category (95% CI [−76.6, −9.4], *p* = 0.012).

**Table 3:** Mixed-effects associations of perceived barriers (PBs) and dietary self-efficacy (DSE) with combined fruit and vegetable intake, fruit intake, and vegetable intake (g/day).

Predictor (PBs/DSE)	Category	Combined fruit and vegetable intake				Fruit intake				Vegetable intake			
		β (g/day)	95% CI	*p*-value	Omnibus *p*-value	β (g/day)	95% CI	p-value	Omnibus *p*-value	β (g/day)	95% CI	p-value	Omnibus *p*-value
PB: Availability	1 (highest barrier)	−53.96	−80.67, −27.26	< 0.001	< 0.001	−44.12	−64.19 to −24.06	< 0.001	< 0.001	2.52	−12.68 to 17.72	0.745	0.419
	2	−40.70	−60.04, −21.36	< 0.001		−46.32	−61.42 to −31.22	< 0.001		5.82	−4.63 to 16.26	0.275	
	3	−48.72	−73.92, −23.52	< 0.001		−47.58	−66.44 to −28.73	< 0.001		2.96	−13.19 to 19.10	0.719	
	4	−38.95	−57.40, −20.50	< 0.001		−47.52	−61.90 to −33.14	< 0.001		8.96	−0.92 to 18.84	0.075	
	5 (ref: lowest barrier)	–	–	–		–	–	–		–	–	–	
PB: Taste	1 (highest barrier)	41.58	12.42, 70.74	0.005	0.025	23.32	1.17 to 45.47	0.039	0.015	6.95	−9.71 to 23.61	0.414	0.029
	2	40.10	15.66, 64.54	0.001		27.62	8.46 to 46.77	0.005		7.27	−6.16 to 20.69	0.288	
	3	38.80	11.76, 65.83	0.005		15.04	−5.72 to 35.80	0.156		17.13	1.53 to 32.74	0.031	
	4	36.46	11.98, 60.94	0.004		21.14	1.98 to 40.30	0.031		13.98	0.53 to 27.44	0.042	
	5 (ref)	–	–	–		–	–	–		–	–	–	
PB: Time	1	20.32	−7.19, 47.83	0.148	0.082	14.37	−5.30 to 34.04	0.152	0.053	5.31	−10.89 to 21.51	0.520	< 0.001
	2	18.25	−1.04, 37.54	0.064		12.92	−2.15 to 27.98	0.093		4.90	−5.72 to 15.51	0.366	
	3	30.73	9.28, 52.18	0.005		−0.26	−16.34 to 15.81	0.974		34.48	21.27 to 47.69	< 0.001	
	4	15.17	−2.48, 32.82	0.092		12.19	−1.50 to 25.87	0.081		5.77	−3.81 to 15.34	0.238	
	5 (ref)	–	–	–		–	–	–		–	–	–	
PB: Cost	1	−21.48	−45.48, 2.52	0.079	0.045	−17.51	−36.32 to 1.30	0.068	< 0.001	−4.91	−17.99 to 8.17	0.462	0.669
	2	−25.31	−46.63, −3.98	0.020		−22.42	−39.35 to −5.48	0.009		−4.10	−15.73 to 7.54	0.490	
	3	−19.87	−46.32, 6.59	0.141		−4.69	−24.04 to 14.67	0.635		−12.33	−28.49 to 3.84	0.135	
	4	−11.99	−33.26, 9.28	0.269		−5.57	−22.28 to 11.15	0.514		−3.90	−15.48 to 7.68	0.509	
	5 (ref)	–	–	–		–	–	–		–	–	–	
PB: Family	1	−14.01	−39.01, 10.99	0.272	0.335	−17.51	−32.58 to −2.44	0.023	0.001	3.39	−11.41 to 18.19	0.653	0.767
	2	−7.54	−25.98, 10.91	0.423		−19.71	−32.08 to −7.35	0.002		7.02	−3.16 to 17.21	0.177	
	3	−21.32	−43.37, 0.73	0.058		−28.42	−42.24 to −14.59	< 0.001		4.83	−8.81 to 18.47	0.488	
	4	−5.72	−22.98, 11.54	0.516		−16.45	−27.68 to −5.23	0.004		4.92	−4.42 to 14.26	0.302	
	5 (ref)	–	–	–		–	–	–		–	–	–	
DSE: Cooking	1 (lowest confidence)	−14.83	−44.56, 14.89	0.328	0.090	−4.34	−25.75 to 17.08	0.692	0.424	−13.36	−29.77 to 3.06	0.111	< 0.001
	2	−7.10	−30.69, 16.49	0.555		−12.80	−31.14 to 5.54	0.171		1.25	−11.52 to 14.02	0.848	
	3	−20.79	−41.43, −0.16	0.048		−11.65	−27.95 to 4.66	0.162		−15.60	−26.57 to −4.62	0.005	
	4 (ref: highest confidence)	–	–	–		–	–	–		–	–	–	
DSE: Taste	1	−43.01	−76.58, −9.44	0.012	0.018	−40.51	−64.96 to −16.06	0.001	0.009	14.38	−3.82 to 32.58	0.121	0.078
	2	−5.87	−32.67, 20.92	0.667		−16.75	−37.27 to 3.77	0.110		18.91	4.57 to 33.24	0.010	
	3	−1.78	−25.71, 22.16	0.884		−16.81	−35.61 to 2.00	0.080		13.94	1.21 to 26.66	0.032	
	4 (ref)	–	–	–		–	–	–		–	–	–	
DSE: Eat Less	1	10.31	−16.28, 36.90	0.447	0.441	5.43	−15.44 to 26.30	0.610	0.114	−0.26	−14.68 to 14.16	0.972	0.054
	2	2.56	−19.39, 24.52	0.819		−12.16	−30.67 to 6.34	0.198		7.39	−4.32 to 19.11	0.216	
	3	−5.64	−26.27, 14.99	0.592		−8.49	−25.93 to 8.95	0.340		−4.09	−15.01 to 6.82	0.462	
	4 (ref)	–	–	–		–	–	–		–	–	–	
DSE: Family Support	1	26.42	−3.53, 56.37	0.084	0.333	8.36	−11.08 to 27.81	0.399	0.808	14.66	−1.59 to 30.90	0.077	0.343
	2	8.95	−15.46, 33.36	0.472		2.10	−15.96 to 20.15	0.820		5.72	−7.57 to 19.01	0.399	
	3	7.64	−14.22, 29.51	0.493		3.98	−12.56 to 20.53	0.637		4.43	−7.35 to 16.22	0.461	
	4 (ref)	–	–	–		–	–	–		–	–	–	
DSE: Canteen	1	9.88	−15.70, 35.45	0.449	0.899	8.73	−6.53 to 23.98	0.262	0.441	−12.49	−26.34 to 1.35	0.077	0.135
	2	5.78	−13.88, 25.45	0.564		9.38	−3.32 to 22.07	0.148		−12.59	−23.28 to −1.90	0.021	
	3	4.88	−13.80, 23.56	0.609		2.97	−6.68 to 12.61	0.547		−8.97	−19.12 to 1.19	0.083	
	4 (ref)	–	–	–		–	–	–		–	–	–	
DSE: Around Me	1	−17.16	−41.48, 7.16	0.167	0.345	9.47	−18.10 to 37.04	0.501	0.815	−6.36	−19.79 to 7.06	0.353	0.003
	2	−12.95	−33.94, 8.05	0.227		10.51	−11.86 to 32.89	0.357		−2.25	−13.80 to 9.29	0.702	
	3	−3.08	−23.37, 17.22	0.766		8.95	−12.38 to 30.28	0.411		12.20	1.08 to 23.32	0.032	
	4 (ref)	–	–	–		–	–	–		–	–	–	

Values represent adjusted β coefficients (g/day) from linear mixed-effects models with random intercepts for participant ID and fixed effects for time and covariates. Omnibus *p*-values reflect the overall significance of each predictor domain. Individual *p*-values correspond to category-specific contrasts relative to the reference group (lowest barrier for PB; highest self-efficacy for DSE). Negative β values indicate lower intake relative to the reference category. PB, Perceived barrier, DSE, Dietary self-efficacy.

Moreover, associations between PBs and intake differed by food groups. For fruit, lower perceived availability was consistently linked to reduced intake (β = −44.1 g/day, 95% CI [−64.2 to −24.1], *p* < 0.001). Family-related PBs were also linked to lower intake across categories, with the largest reduction observed in the “neither agree nor disagree” group. Cost-related barriers were similarly associated with lower fruit, although effects were weaker and inconsistent. Consistent with combined FV findings, taste-related PBs showed a positive association with fruit intake, with higher reported barriers corresponding to greater consumption. For vegetables, few predictors demonstrated statistically meaningful associations. Time-related PBs exhibited a non-monotonic pattern, with only the intermediate category (“neither agree nor disagree”) reporting the significantly higher intake than the lowest-barrier reference group (β = 34.5, 95% CI [27.3, 47.7], *p* < 0.001). Taste-related PBs were positively associated only in mid-level PB categories.

In DSE analyses, lower confidence in adapting to different tastes was linked to substantially lower fruit intake (β = −40.5 g/day, 95% CI [−65.0, −16.1], *p* = 0.001). For vegetables, cooking-related self-efficacy showed a smaller, non-monotonic association, with lower intake observed in one lower-confidence group relative to the highest-confidence group (β = −16.0 g/day, 95% CI [−26.6, −4.6], *p* = 0.005). In contrast, lower confidence in resisting social influence was associated with slightly higher vegetable intake in one category.

Sensitivity analyses were performed excluding the 9-year data (Supplementary Table 3). The overall trends remained broadly consistent with the primary models, with availability, cost, and taste-related PBs, as well as taste- and cooking-related DSE, showing similar directions of association, although statistical significance was not consistently retained across all predictors and outcomes.

## Discussion

Perceived availability and cost barriers were the most consistent factors linked to lower fruit and vegetable (FV) intake over 9 years, while dietary self-efficacy (DSE) played a comparatively minor role. Although these psychosocial measures were subjective and constrained by measurement limitations, the findings suggest that long-term FV intake in this cohort is more closely associated with structural constraints as experienced by individuals, rather than individual motivation or confidence alone. Persistently low FV intake, remaining well below recommended levels across all time points, signals a substantial public health challenge and underscores the urgency of addressing the upstream determinants of dietary behavior.

Objective evidence from India and other low- and middle-income countries (LMICs) strongly corroborates the structural constraints reflected in perceived availability and cost barriers. Food price inflation, supply-chain inefficiencies, and inadequate cold-storage infrastructure continue to restrict access to fresh produce and contribute to intra-year price volatility (44, 45). In Kerala, dependence on local retail markets may further increase vulnerability to these pressures (11, 46). Although FV intake in Kerala may be higher than in other parts of India, it remains suboptimal, suggesting persistent gaps despite favorable conditions (47). The concordance between self-reported barriers and documented food-system limitations suggests that perceived constraints stem from structural realities rather than temporary individual concerns. Because these system-level constraints are widespread across many LMIC contexts, the findings are likely to have relevance beyond this population (48). Within such settings, improving access and affordability is likely to be fundamental to increasing FV intake; interventions that do not address these structural constraints may have limited impact.

Although several predictors of perceived barriers (PBs) were consistent with prior literature, notable divergences were observed. Unlike findings from other regions of India (8, 49) and from other LMICs (25, 26, 50), time- and social-related barriers were not independently associated with FV intake in this cohort. These differences may reflect context-specific factors in Kerala, such as its comparatively advanced epidemiological and health system profile, which may reduce the salience of time constraints and social influences on dietary behavior (17, 19, 51). Moreover, the high-risk nature of the sample may be associated with greater health consciousness and motivation to adopt dietary recommendations, potentially attenuating the apparent influence of these barriers relative to persistent structural limitations. These findings remain exploratory and warrant further investigation in similar settings.

Against the backdrop of the structural and perceptual constraints, DSE showed modest, food-specific associations with intake. For fruit, markedly low confidence in adapting to different tastes was linked to reduced consumption. This pattern aligns with self-efficacy theory, which proposes that the effects of confidence on behavior are often non-linear; with the largest behavioral gains occurring as efficacy moves from very low to higher levels, followed by diminishing returns beyond that point (52, 53). This non-linear finding supports modeling these constructs as categorical variables, rather than assuming a linear trend across Likert scale categories. For vegetables, associations with cooking confidence and resistance to social influence were present but mostly weak or inconsistent. Taken together, this limited independent role of DSE aligns with evidence indicating that self-efficacy alone rarely produces meaningful, lasting change in dietary behavior without supportive environments (54–56). Although stronger effects of DSE have been reported among Indian adolescents (57), this contrast likely reflects age- and context-specific factors, including structured settings and peer norms that amplify responsiveness to self-efficacy-based strategies. Social Cognitive Theory emphasizes that self-efficacy drives behavior change only when conditions support the intended action (53). In contexts characterized by persistent affordability and availability constraints, the independent impact of DSE on dietary behavior is therefore likely to be limited.

An additional insight was the presence of counterintuitive associations between PBs and intake. Contrary to evidence from other LMICs and high-income settings (8, 58), higher perceived taste-related barriers were associated with greater intake across food groups. These findings should be interpreted cautiously and may reflect complex relationships between perception and behavior. It is possible that dietary behaviors influence PBs, rather than the reverse, highlighting the potential for bidirectional relationships. For example, individuals with higher intake may be more aware of taste-related challenges due to greater exposure, or these perceptions may reflect unmeasured factors such as health awareness or engagement with dietary change following the intervention. These findings are hypothesis-generating and warrant further investigation in studies specifically designed to examine underlying mechanisms.

Extending this complexity, moderate or neutral perceptions of certain barriers warrant closer attention. Vegetable intake was highest among participants who selected “neither agree nor disagree” for time-related PBs, and fruit intake peaked among those in the same neutral category for family-related PBs. These findings suggest that ambiguous or moderate perceptions of barriers may be associated with greater behavioral flexibility, whereas individuals at the extremes may face either genuine constraints or hold overconfident expectations that do not translate into actual behavior. Higher intake among those reporting mid-range barriers may also reflect more favorable socioeconomic conditions and access to resources that attenuate the practical impact of perceived obstacles (53, 59). Although this phenomenon remains underexplored in Indian contexts, such moderate perceptions may reflect adaptive responses within constrained environments.

Fruit and vegetable intake exhibited distinct trajectories over the 9-year period, with fruit consumption declining and vegetable intake increasing. These contrasting patterns may reflect differential responses to price fluctuations and inconsistent availability during the study period (5, 10, 44, 45). Fruit, typically consumed as a standalone and discretionary item, may be more vulnerable to access constraints and affordability pressures. In contrast, vegetables constitute a core component of daily meals in South India and can be substituted across low-cost varieties or incorporated into multiple dishes. Such structural and cultural factors may help buffer vegetable intake against supply and price volatility, while amplifying the vulnerability of fruit consumption.

Building on these differential external influences, psychosocial factors also appeared to operate distinctly across food groups. Availability and family-related barriers were most strongly linked to lower fruit consumption but were not independently associated with vegetable intake, whereas cooking-related self-efficacy showed a significant, non-monotonic association with vegetable intake. This pattern suggests food-specific behavioral pathways: fruit intake, being more discretionary, may be more sensitive to sensory expectations and thus more closely linked to taste-related confidence, while vegetable intake is embedded within habitual meals and preparation routines, making it more strongly tied to technical and cognitive capacities such as cooking skills and planning ability (60, 61). Prior evidence similarly indicates that sensory factors are more strongly tied to fruit intake, whereas functional competencies feature more prominently in relation to vegetable consumption (32, 61). Recent reviews similarly emphasize the importance of examining fruit and vegetable consumption as related but distinct behaviors with unique determinants (61, 62), reinforcing the value of food-specific public health strategies that target the most salient perceptual and psychosocial mechanisms.

A major strength of this study is its longitudinal design, which enabled assessment of within-person changes and provided robust estimates across a large, well-characterized cohort with high follow-up retention. The repeated measurement of psychosocial and behavioral factors strengthens internal validity relative to cross-sectional designs by allowing the assessment of temporal associations.

Nevertheless, several limitations warrant consideration. Given the observational and secondary nature of the study, the findings reflect longitudinal associations rather than causal effects, and the directionality of relationships between psychosocial factors and dietary behaviors cannot be established.

A key methodological limitation is the change in dietary assessment at the 9-year follow-up, where a simplified serving-based measure replaced the detailed FFQ used in earlier waves. To enable comparability across waves, responses were harmonized to a common metric; however, this limits the interpretation of absolute intake over time and introduces potential non-differential measurement error. Consequently, temporal trends, particularly the decline in fruit intake, should be interpreted with caution, as some observed differences may reflect measurement variation rather than true behavioral change. The primary inference therefore relies on the relative longitudinal associations between PBs, DSE, and intake, under the assumption that measurement error is broadly non-differential across exposure groups. Sensitivity analyses excluding the 9-year data yielded consistent results, supporting the robustness of these associations.

All dietary data were self-reported and subject to recall and social desirability bias, which may lead to systematic under- or overestimation of intake (63, 64). In addition, psychosocial measures were based on Likert-type items, which are vulnerable to response biases such as central tendency and acquiescence, and provide perception-based rather than objective indicators of behavioral capabilities (65, 66). Although culturally adapted for the study context, these scales have not undergone formal psychometric validation; therefore, construct validity cannot be fully assured. The PB domains were assessed using single-item measures, which were designed to capture conceptually distinct and commonly reported barriers to healthy eating, providing some support for their face and content validity. However, this approach precluded assessment of internal consistency and may not fully capture the multidimensional nature of PBs, with implications for construct validity. Conversely, the high internal consistency observed for the DSE scale (Cronbach's α = 0.949) may be partly explained by its domain-specific focus but may also reflect some degree of item redundancy and limited coverage of the broader construct. Together, these factors may introduce non-differential measurement error and misclassification of psychosocial exposures, potentially biasing associations toward the null and underestimating the strength of the observed relationships between psychosocial factors and dietary intake. Additionally, the simplified categories, used in descriptive analyses (e.g., “high” vs. “low”) for improving interpretability, may obscure more nuanced variation within constructs.

Furthermore, the generalisability of these findings is limited. The study population comprised adults from Kerala who were at high risk of type 2 diabetes and had a high prevalence of overweight and obesity. Consequently, behavioral patterns and responses to PBs observed in this cohort may differ from those of the general population. The findings may therefore not be directly applicable to the broader Indian population, younger age groups, or high-income settings characterized by different food environments. Nonetheless, the results may be more relevant to comparable LMIC contexts undergoing nutritional and epidemiological transitions.

Finally, some confidence intervals were wide, reflecting heterogeneity and small subgroup sizes; accordingly, effect estimates should be interpreted with caution even when directionally consistent with study hypotheses. Although analyses were guided by *a priori* hypotheses, the absence of formal correction for multiple comparisons may increase the risk of type I error. As comparisons were structured around predefined constructs, findings- particularly weaker or non-monotonic associations- should be interpreted with caution.

These findings have potential implications for intervention design and public health policy. Rather than relying solely on stand-alone behavior change strategies, effective approaches may prioritize structural interventions that directly address affordability and access, including investments in cold-chain infrastructure, transportation networks, and local procurement systems to reduce spoilage and improve supply efficiency. Financial incentives, such as subsidies or cashback schemes for FV purchases, have shown emerging effectiveness in rural India and may warrant broader evaluation (67). These system-level approaches could be integrated with targeted education addressing key PBs, for example, promoting cost-effective purchasing and food-storage practices to reduce waste and financial burden. Where these enabling conditions are limited, interventions focused solely on information provision or self-efficacy enhancement may be less likely to achieve meaningful and sustained change in diet quality.

Future research should examine whether DSE functions as a mediating or buffering construct in the relationship between PBs and dietary behavior, particularly in light of the complex and sometimes counterintuitive associations observed in this study. Further investigation into these patterns may enhance understanding of compensatory behaviors in resource-constrained environments. Stratified analyses by sociodemographic factors would also improve understanding of heterogeneity in response to barriers and inform culturally relevant strategies to reduce dietary inequities.

## Data Availability

The data analyzed in this study is subject to the following licenses/restrictions: The K-DPP dataset is subject to ethical and confidentiality restrictions and is not publicly available. Access requires approval from the K-DPP investigators and relevant ethics committees, and may be granted to qualified researchers upon reasonable request and execution of a data-sharing agreement. Requests to access these datasets should be directed to Tilahun Haregu, Tilahun.Haregu@baker.edu.au.
