# Supplementary material for: Perceived barriers to healthy eating and self-efficacy among Indian adults at high risk of type 2 diabetes: a secondary analysis of the K-DPP study
Source: Front Nutr. 2026 May 25;13:1777691. doi: 10.3389/fnut.2026.1777691 (PMC13243027; doi:10.3389/fnut.2026.1777691)
Supplement: Supplementary file 1 [file Table_1.docx]

Supplementary Material

**Supplementary Table 1: Key independent and dependent variables**

| **Variable type** | **Variable** | **Description/measurement [variable ID]** |
| --- | --- | --- |
| **Independent** | Perceived barriers (PBs) | Self-reported survey items on perceived factors for healthy eating recorded on a 5-point Likert scale (1 = Strongly agree to 5 = Strongly disagree)  *For each statement about healthy eating (a diet low in sugar or fried foods, or high in fruit and vegetables), please tell me to what extent you agree:*   - The lack of availability of healthy food makes it difficult to have a healthy diet [Availability], - Healthy food does not taste as good as unhealthy food [Taste], - I do not have the time to eat healthily [Time], - The cost of healthy food makes it difficult to have a healthy diet [Cost], and - People in my family would not want me to make or eat healthy food [Family]. |
|  | Dietary  self-efficacy (DSE) | Self-reported survey items on dietary self-efficacy reported on a 4-point Likert scale (1 = Very uncertain to 4 = Very certain)  *I can manage to stick to healthier eating habits (e.g. fewer fried foods, or lower in sugar, or more fruit and vegetables):*   - even if it means changing the ways of cooking in my family (confidence in modifying cooking methods [Cooking]), - even if some foods taste different at first (adapting to different tastes [Taste]), - even if I have to eat less and say “no” to food offered during special events and festivities (refusing unhealthy foods at social events [Eat_less]), - even if I do not receive a great deal of support for healthy eating from my family or friends (maintaining healthy eating despite lack of family or peer support [Family_support]), - even if the canteen at workplace / restaurant does not serve healthy food (managing when healthy options are unavailable at work or restaurants [Canteen]), and - even if others around eat unhealthy food (resisting the influence of others consuming unhealthy foods [Around_me]). |
| **Dependent** | Fruit and vegetable intake | Grams consumed per day, based on self-reported/estimated dietary recall.   - Fruit intake [fruit intake] - Vegetable/tuber intake [vegetable intake] - Combined intake [FV intake] |
| **Control** | Age, gender, marital status, education level, monthly expenditure, physical activity (leisure), sleep duration, sedentary hours, family history of diabetes, cluster | |

**Supplementary Table 2: Significant influences of perceived barriers and dietary self-efficacy on fruit and vegetable intake at baseline**

|  | Variable | Categories | Fruit  Median (IQR) | p-value | Veg/Tuber  Median (IQR) | p-value |
| --- | --- | --- | --- | --- | --- | --- |
| Perceived Barriers | Availability | High barrier | 99.2 (126.9) | 0.841 | 102.9 (85.7) | 0.176 |
|  |  | Low barrier | 95.4 (127.7) |  | 102.9 (88.8) |  |
|  |  | Neither | 97.1 (118.5) |  | 42.7 (154.3) |  |
|  | Taste | High barrier | 99.9 (137.9) | 0.034 | 97.1 (85.7) | 0.007 |
|  |  | Low barrier | 88.1 (110.3) |  | 114.3 (86.1) |  |
|  |  | Neither | 106.4 (135.8) |  | 122.7 (97.1) |  |
|  | Time | High barrier | 97.3 (155.6) | 0.393 | 97.1 (92.0) | 0.088 |
|  |  | Low barrier | 96.8 (122) |  | 102.9 (88.8) |  |
|  |  | Neither | 80.0 (144.9) |  | 114.3 (80.4) |  |
|  | Cost | High barrier | 83.0 (109.0) | <0.001 | 102.9 (91.4) | 0.407 |
|  |  | Low barrier | 106.1 (135.1) |  | 102.9 (88.8) |  |
|  |  | Neither | 146.9 (147.5) |  | 102.9 (125.7) |  |
|  | Family | High barrier | 83.0 (120.4) | 0.239 | 97.1 (80.0) | 0.005 |
|  |  | Low barrier | 98.5 (127.5) |  | 114.3 (88.8) |  |
|  |  | Neither | 104.1 (119.3) |  | 100.0 (117.0) |  |
| Dietary Self-Efficacy | Cooking | Lower DSE | 98.6 (140.1) | 0.984 | 102.9 (85.7) | 0.588 |
|  |  | Higher DSE | 96.0 (121.1) |  | 102.9 (88.8) |  |
|  | Taste | Lower DSE | 84.9 (136.1) | 0.123 | 102.9 (84.4) | 0.552 |
|  |  | Higher DSE | 97.4 (121.9) |  | 102.9 (90.1) |  |
|  | Eat_less | Lower DSE | 100.5 (145.1) | 0.582 | 97.1 (85.7) | 0.313 |
|  |  | Higher DSE | 95.3 (117.9) |  | 102.9 (88.8) |  |
|  | Family_support | Lower DSE | 88.4 (145.0) | 0.123 | 97.1 (82.0) | 0.114 |
|  |  | Higher DSE | 98.6 (117.4) |  | 102.9 (88.8) |  |
|  | Canteen | Lower DSE | 96.9 (141.2) | 0.945 | 102.9 (84.4) | 0.122 |
|  |  | Higher DSE | 96.7 (114.0) |  | 102.9 (88.8) |  |
|  | Around_me | Lower DSE | 94.6 (139.2) | 0.612 | 97.1 (81.3) | 0.054 |
|  |  | Higher DSE | 96.9 (118.7) |  | 102.9 (88.8) |  |

**Note:** Values are presented as median (interquartile range, IQR). For perceived barriers, group differences in fruit and vegetable/tuber intake were assessed using Kruskal-Wallis tests across three perceived barrier (PB) categories: *High barrier* (*Strongly agree/Agree*), *Low barrier* (*Strongly disagree/Disagree*), and *Neither* (*Neither agree nor disagree*). Intake values represent grams per day, derived from self-reported food frequency questionnaire data at baseline. Statistical significance was set at *p* < 0.05. For dietary self-efficacy, group differences in fruit and vegetable/tuber intake were assessed using Mann-Whitney U tests (MW) across two dietary self-efficacy (DSE) levels: *Lower DSE* (*Very uncertain/Rather uncertain*) and *Higher DSE* (*Rather certain/Very certain*). Intake values represent grams per day, derived from self-reported food frequency questionnaire data at baseline. Statistical significance was set at *p* < 0.05.

**Supplementary Table 3: Sensitivity Analysis without 9-year data - Associations of perceived barriers (PBs) and dietary self-efficacy (DSE) with fruit intake and vegetable intake (g/day)**

|  | Variable | Combined Fruit and Vegetable Intake | | | | Fruit Intake | | | Vegetable Intake | | |
| --- | --- | --- | --- | --- | --- | --- | --- | --- | --- | --- | --- |
|  |  | Denominator df | F | p-value | Denominator df | | F | p-value | Denominator df | F | p-value |
| Perceived Barriers | Availability | 2356.670 | 2.349 | 0.052 | 2320.328 | | 6.614 | <0.001 | 2556.927 | 0.887 | 0.471 |
|  | Taste | 2270.022 | 3.625 | 0.006 | 2245.618 | | 3.899 | 0.004 | 2433.905 | 4.447 | 0.001 |
|  | Time | 2329.701 | 0.525 | 0.717 | 2301.378 | | 1.237 | 0.293 | 2522.349 | 1.468 | 0.209 |
|  | Cost | 2378.111 | 3.098 | 0.015 | 2342.190 | | 2.146 | 0.073 | 2591.141 | 1.400 | 0.232 |
|  | Family | 2370.517 | 0.621 | 0.647 | 2340.508 | | 1.184 | 0.316 | 2552.645 | 0.730 | 0.572 |
| Dietary Self-Efficacy | Cooking | 2336.548 | 4.366 | 0.005 | 2353.332 | | 3.186 | 0.023 | 2361.191 | 4.700 | 0.003 |
|  | Taste | 2391.024 | 1.254 | 0.289 | 2382.065 | | 4.646 | 0.003 | 2487.315 | 2.137 | 0.094 |
|  | Eat_less | 2251.950 | 1.498 | 0.213 | 2222.860 | | 1.223 | 0.300 | 2473.109 | 0.503 | 0.680 |
|  | Family_support | 2375.822 | 0.712 | 0.545 | 2354.687 | | 1.064 | 0.363 | 2518.736 | 0.474 | 0.700 |
|  | Canteen | 2251.345 | 0.269 | 0.848 | 2186.823 | | 1.697 | 0.166 | 2546.247 | 0.934 | 0.423 |
|  | Around_me | 2364.221 | 0.537 | 0.657 | 2335.533 | | 0.653 | 0.581 | 2529.072 | 2.003 | 0.111 |

Note: Estimates are from linear mixed-effects models with random intercepts for participants. Models adjust for sociodemographic and lifestyle covariates, as well as intervention arms. PB and DSE variables were treated as categorical predictors. Type III Wald F-tests evaluate overall associations with fruit and vegetable intake (g/day). The 9-year wave was excluded as a sensitivity analysis.
